# Different methods for assessing glomerular filtration rate in the elderly

**DOI:** 10.1590/1806-9282.20221101

**Published:** 2024-01-26

**Authors:** Gilsirene Scantelbury de Almeida, Noeli das Neves Toledo, Miharu Maguinoria Matsuura Matos, Luis Cuadrado Martin, Roberto Jorge da Silva Franco

**Affiliations:** 1 Universidade Federal do Amazonas, Manaus School of Nursing – Manaus (AM), Brazil.; 2 Universidade Federal do Amazonas, Getúlio Vargas University Hospital – Manaus (AM), Brazil.; 3 Universidade Estadual Paulista “Júlio de Mesquita Filho”, Faculty of Medicine – Botucatu (SP), Brazil.

**Keywords:** Glomerular filtration rate, Cystatin C, Chronic kidney disease

## Abstract

**OBJECTIVE:**

The objective of this study was to identify the best method to replace cystatin C in the evaluation of glomerular filtration in the elderly.

**METHODS:**

Individuals over 60 years of age from a primary care center were studied. Blood was collected to determine creatinine and cystatin C and 24-h urine. Three methods were compared to determine glomerular filtration: Creatinine clearance, Cocroft-Gault, modification of diet in renal disease, and Collaboration Epidemiology of Chronic Kidney Disease based on creatinine, considering as a reference the determination of glomerular filtration using the cystatin-based Chronic Kidney Disease Epidemiology Collaboration equation. The statistical methods used were linear regression, Bland-Altman curve, and receiver operating characteristic.

**RESULTS:**

A total of 180 elderly people were evaluated, but 14 patients were lost from the sample, resulting in a total of 166 patients. The average age of patients was 66.9±6.1 years, and 69.8% were females. Regarding the number of patients eligible for the study, there were 12 black, 108 brown, and 46 white, 42.77% hypertensive, and 38.3% diabetic. Glomerular filtration was less than 60 mL/min in 22.28% of patients. Regarding the evaluation of the different equations, the correlation coefficient was lower for creatinine clearance and progressively higher for Cocroft-Gault, modification of diet in renal disease, and Collaboration Epidemiology of Chronic Kidney Disease based on creatinine. The Bland-Altman diagram and the receiver operating characteristic curve showed similar performance to the correlation coefficient for the different equations evaluated.

**CONCLUSION:**

Collaboration Epidemiology of Chronic Kidney Disease based on creatinine presented the best performance. Creatinine debug had the worst performance, which reinforces the idea that 24-h urine collection is unnecessary in these patients.

## INTRODUCTION

In Brazil, it is estimated that about 20 million people have chronic kidney disease (CKD), which is strongly associated with morbidity and mortality^1^. The diagnosis of CKD should be based on the persistence, for 3 months or more, of a glomerular filtration rate (GFR) £60 mL/min or on the structural or functional abnormality of the kidney, which is demonstrated by pathological changes or by markers of renal injury, even if abnormalities are present as assessed by blood, urine, or imaging tests. A chronically low GFR (<60 mL/min/1.73 m^2^) is sufficient to make the diagnosis of CKD, with or without other markers of kidney damage^2^.

The cystatin-based Chronic Kidney Disease Epidemiology Collaboration equation (CKD-EPI-Cyst) is based on the creatinine and cystatin C levels. The lysosomal protein cysteine is particularly attractive as a marker of kidney function^3^. The use of cystatin C may be particularly advantageous in elderly patients, as it is the method with the best validation in this age group^4^. However, the dosage of this substance is still unfeasible in most centers due to its high price and the lack of availability of this test in the Unified Health System. This fact leads to the search for alternatives to cystatin C. Evaluating whether GFR estimation equations based only on creatinine could be used instead of the equation that adopts cystatin C and verifying whether these equations can estimate the GFR obtained by cystatin C are of practical interest, especially in places with limited financial resources.

Therefore, the aim of this research was to identify the best method to replace cystatin C in the assessment of GFR in the elderly. Equations based on serum creatinine levels were compared, and the equation based on cystatin C were adopted as reference.

## METHODS

This is a cross-sectional study to verify the diagnostic performance of different methods of glomerular filtration assessment in an elderly population sample. Four methods were compared, namely, Cockroft-Gault (C&G), modification of diet in renal disease (MDRD), Collaboration Epidemiology of Chronic Kidney Disease based on creatinine (CKD-EPI-Cr), and creatinine release (Cr). GFR assessed by CKD-EPI-Cyst was adopted as the standard. Although inulin is considered the ideal marker of GFR since it is freely filtered by the glomerulus and is not reabsorbed or secreted by the renal tubule, direct measurement of GFR using inulin is not feasible because it requires continuous intravenous infusion and a fixed time for urine collection. Due to age-related limitations, the high prevalence of prostatic disease in the elderly leads to inaccuracies in 24-h urine volume and the possible presence of residual volume. We adopted CKD-EPI-Cyst as the standard of analysis.

Data collection was carried out at the Center for Integrated Care for the Elderly (CAIMI), a service center created to provide care exclusively to individuals aged 60 years and above. CAIMI is an interdisciplinary care service center that involves in activities in the areas of social work, nursing, pharmaceutics, laboratory, and medical assistance. They are distributed throughout the city of Manaus-AM in the health districts located in all directions. The CAIMI located in the western district was chosen to conduct the research because it is the most representative of the target population. Patients aged above 60 years who sought general clinical care for the first time were included. The age group chosen was due to the orientation made by the National Health Policy of the Elderly Person in Brazil, in line with the principles and guidelines of the Unified Health System. These consider Brazilians aged 60 years or older to be elderly^5^. Patients who sought health care at this CAIMI from September to November 2012 were included sequentially. Patients from other specialties with suspected kidney problems and older adults who were unable to answer the questionnaire due to cognitive limitations and unaccompanied were excluded. Creatinine and cystatin levels were measured in a single blood sample.

Weight and height measurements were carried out using an electronic scale and a portable stadiometer, respectively. The elderly were weighed standing with barefoot and wearing light clothing. The tests were collected in the morning with fasting for 12 h. Blood samples were stored at −80°C. A 24-h urine collection was performed the day before the blood test. The tests were performed using commercial kits from Winner on an automated BT 3000 plus equipment from Winner Lab, Rome, Italy. Samples were processed for serum and urine creatinine and serum cystatin C assay. Cystatin C was measured by immunoturbidimetry. The equations used in the study were as follows:

C&G equation

GFR=[(140−age in years)×Weight(kg)/Cr(mg/dL)×72]×0.85 (female) or 1.0 (male) (mL/min)^6^.

The value was adjusted for body surface area using the Dubois & Dubois formula (mL/min/1.73 m^2^)^7^.

MDRD equation

$\text{GFR}=186\times \left[\text{Cr}\left(\text{in mg}/\text{dL}\right)\right]-1.15\times \left[\text{age}\left(\text{years}\right)\right]-0.203\times \left[0.742\left(\text{if female}\right)\times [1.212\left(\text{if black}\right)\right]\left(\text{mL}/\min/1.73{\text{m}}^{2}\right)$ ^8^.

CKD-EPI-Cr 2009 equation^9^

Correcting for sex of serum creatinine (mg/dL) in case of female ≤0.7, the estimated GFR equation is 144×(Creat/0.7)–0.329×0.993 years [×1159 if black] or ≥0.7 144×(Creat/0.7)–1209×0.993 age [×1159 if black]. In men ≤0.9, the estimated GFR equation is 144×(Creat/0.9)–0.401×0.993 age [×159 if black] or ≥0.9 144×(Creat/0.9)–1.209×0.993 age [×159 if black].

CKD-EPI-Cyst 2012 equation^9^

Correcting for sex of serum creatinine (mg/dL) being female ≤0.7 and cystatin C (mg/L) ≤0.8, the estimated GFR equation is 130×(Creat/0.7)–0.248×(Cys/0.8)–0.375×0.995 age [××1.08 if black] or if ≤0.7 and cystatin C (mg/L)>0.8, the estimated GFR equation is 130×(Creat/0.7)–0.248×(Cys/0.8)–0.711×0.995 [×1.08 if black]. However, if serum creatinine (mg/dL)>0.7 and cystatin C (mg/L)≤0.8 130×(Creat/0.7)–0.601×(Cys/0.8)–0.375×0.995 age [×1.008 if black] or if serum creatinine (mg/dL)>0.7 and cystatin C (mg/L)>0.8130×(Creat/0.7)–0.601×(Cys/0.8)–0.711×0.995 age [×1.08 if black].

In men, creatinine (mg/dL) ≤0.9 and cystatin C (mg/L) 0.8, the estimated GFR equation is 135×(Creat/0.9)–0.207×(Cys/0.8)–0.375×0.995 age [×01.08 if black] or if ≤0.9 and cystatin C (mg/L)>0.8 135×(Creat/0.9)–0.207×(Cys/0.8)–0.711×0.995 age [×1.08 if black]. However, if creatinine (mg/dL)>0.9 and cystatin C (mg/L), the estimated glomerular filtration rate equation is 135 × (Creat/0.9)≤0.8–0.601×(Cys/0.8)–0.375×0.995age [×1.08 if black] or if Creatinine (mg/dL)>0.9 and Cystatin C (mg/L)>0.8135×(Creat/0.9)–0.601×(Cys/0.8)–0.711×0.995 age [×1.08 if black].

### Statistical analysis

The sample consisted of 180 elderly people, sufficient to detect a correlation coefficient of 0.25 with a beta error of 0.2 and an alpha error of 0.05. Data were presented as mean±standard deviation. Qualitative variables were described as absolute frequency and percentage. Glomerular filtration values by the different methods were compared with the standard (CKD-EPI-Cyst). To compare the methods, scatterplots were constructed, and the correlation coefficient, as well as Bland Altman diagrams and receiver operating characteristic (ROC) curves, was calculated. For statistical inferences, the significance level (p<0.05) was considered. Statistical analyses were performed using SPSS 21.0. This research was approved by the Research Ethics Committee of the Federal University of Amazonas, under number 0261.0115.000-10.

## RESULTS

A total of 180 elderly people were examined. However, there was a sample loss of 14 individuals, and of the 166 eligible, there were no patients with clinically manifest cancer in this sample. Socio-demographic and clinical data are described in Table 1.

**Table 1:** Socio-demographic and clinical data of the elderly at the Center for Integrated Care for the Elderly in Manaus/Amazonas/Brazil.

Variables		Number	%
Age		66.9 (6.1)	
Sex			
	Men	50	30.1
	Women	116	69.8
Race and color			
	White	46	27.71
	Black	12	7.22
	Brown	108	65.07
Hypertensive		71	42.77
	Women	50	30.12
	Men	21	12.65
Kidney disease		34	20.48
Diabetes		69	38.3
Body mass index		28.07 (4.7)	27.67 (18.81–46.34)
	Low weight	17	10.24
	Suitable weight	60	36.14
	High weight	103	62.04
Medicines used			
	Antihypertensive	93	56.02
	Antiglycemic	33	19.88
	Did not use medications	40	24.10
Glomerular filtration rate			
	Internship I (TFGe>90)	33	19.88
	Internship II (TFGe between 90 and 60)	96	57.84
	Internship IIIa (TFGe between 60 and 45)	24	14.45
	Internship IIIb (between 45 and 30)	13	7.83
	Internship IV (between 30 and 15)	0	0
	Internship V (<15)	0	0

### Evaluation of the Cockcroft-Gault equation

**Figure 1:**
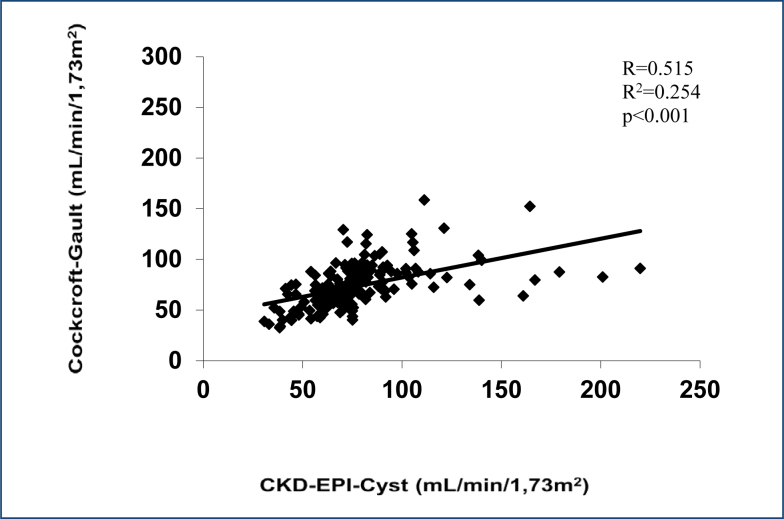
Scatter diagrams, Bland-Altman, and receiver operating characteristic curves for the comparison of Cockroft-Gault with the reference of the equation of cystatin-based Chronic Kidney Disease Epidemiology Collaboration.

The comparison of the determination of glomerular filtration by the Cockcroft-Gault formula with glomerular filtration determined by the CKD-EPI-Cyst equation is presented in Figure 1. Pearson's correlation coefficient was 0.515 (p<0.001), and r^2^ was 0.254.

### Evaluation of the modification of diet in renal disease equation

**Figure 2:**
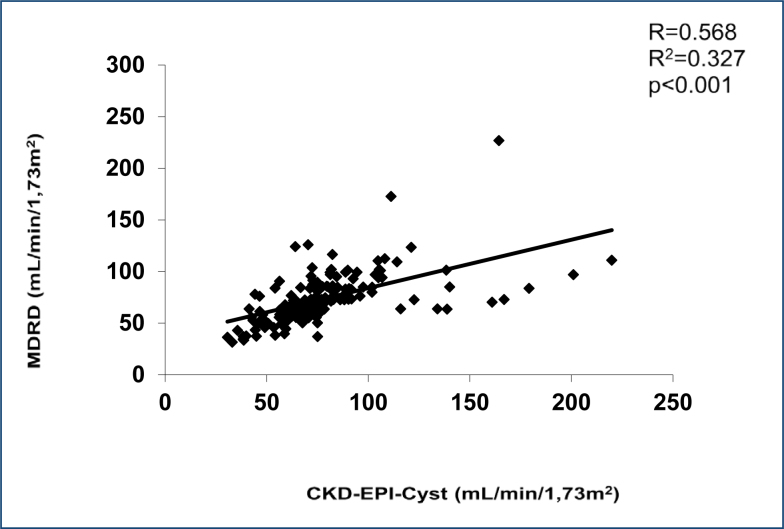
Scatter diagrams, Bland-Altman, and receiver operating characteristic curves for the comparison of modification of diet in renal disease from Collaboration Epidemiology of Chronic Kidney Disease based on creatinine with the reference of the equation of cystatin-based Chronic Kidney Disease Epidemiology Collaboration.

The comparison of the determination of glomerular filtration by the MDRD equation with glomerular filtration determined by the CKD-EPI-Cyst equation is shown in Figure 2. A Pearson correlation coefficient of 0.568 (p<0.001) and an r^2^ of 0.327 were observed.

### Evaluation of the Collaboration Epidemiology of Chronic Kidney Disease based on creatinine equation

**Figure 3:**
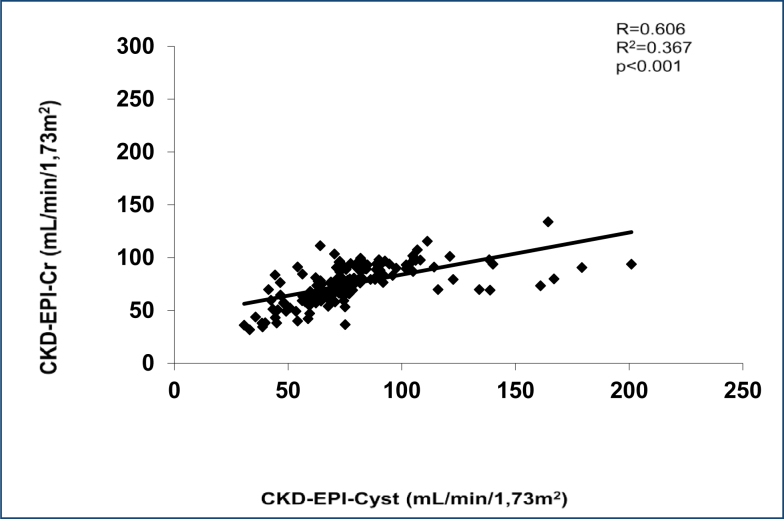
Scatter diagrams, Bland-Altman, and receiver operating characteristic curves for the comparison Collaboration Epidemiology of Chronic Kidney Disease based on creatinine from CKD-EPI-Cys with the reference of the equation of cystatin-based Chronic Kidney Disease Epidemiology Collaboration.

The comparison of glomerular filtration determined by the CKD-EPI-Cr equation with glomerular filtration determined by the CKD-EPI-Cyst equation is shown in Figure 3. A Pearson correlation coefficient of 0.606 (p<0.001) (Figure 1) and an r^2^ of 0.367 were observed.

## DISCUSSION

Chronic kidney disease is a worldwide public health problem that mainly affects the elderly. The utilization of creatinine dosage equations has become the most common method for evaluating glomerular filtration^10^. Thus, this study aimed to identify the best method to replace cystatin C in the evaluation of glomerular filtration in the elderly. It was observed that the GFR evaluated by the CKD-EPI-Cr formula was the one that was closest to the adopted standard.

Creatinine is the endogenous marker most commonly used in clinical practice, either by applying equations based on its serum determination or in conjunction with 24-h urine collection for CrCl determination. The latter method also has several disadvantages. These include the difficulty of collecting 24-h urine^11^.

The National Kidney Foundation guideline recommends “not using creatinine alone as a method of assessing the level of renal function.” A practical clinical solution to the problem is to use creatinine to estimate GFR using equations that include parameters correlated with muscle mass, namely, age, gender, ethnicity, and body weight^11^. Several equations, such as CocG, MDRD, CKD-EPI-Cr^12^, and CKD-EPI-Cyst^13^, endorsed by the National Kidney Foundation have been proposed. The CocG equations, like the MDRD, underestimate GFR in populations with high GFR levels, such as type 1 diabetics without microalbuminuria and kidney transplant donors^14^. In this study, they are in line with the literature, as can be seen in the Bland-Altman diagram. These equations have not been validated for children, older adults over 70 years, pregnant women, or other demographic subgroups. Studies using the established gold standard have concluded that the MDRD equation appears to be more accurate than the CocG equation^15^. We identified this finding in the study; therefore, the performance assessment parameters of MDRD were better than CocG when compared with CKD-EPI-Cyst.

The MDRD equation is thought to underestimate GFR in individuals with normal or increased GFR, but as is clear from the Bland-Altman diagram in this study, there was no association between GFR level and deviations from the standard used. The equation that considers race in the calculation of glomerular filtration was used, since at the time of this study, however, the CKD-epi equation that disregards this demographic variable had not been developed.

The definition of race in the Brazilian population is made difficult by the high degree of miscegenation, but this limitation of our study tends to underestimate our findings and increase the dispersions observed. Therefore, if we could define the race of our patients absolutely, our results would be better. Thus, despite the uncertainty regarding the definition of race, we obtained positive data.

In the regression between GFR assessed by CKD-EPI-Cyst and GFR assessed by ClCr, we observed that there is a statistically significant correlation. However, there is a marked dispersion of the points. When CKD-EPI-Cyst was zero, ClCr was 62.1 mL/min/1.73 m^2^, which characterizes an overestimation of the real GFR value, making this method of GFR assessment unfeasible in this population. Our results support the idea that the usefulness of GFR using CrCl with 24-h urine collection should be re-evaluated in medical practice.

Validation of the CocG equation was based on hospitalized men aged 18–92 years with normal renal function as the basis for estimating creatinine clearance. It was not standardized to a body surface area of 1.73 m^2^ and was corrected for women. It underestimates GRF because tubular creatinine secretion and weight gain due to obesity or fluid overload are not taken into account^16^. These observations are consistent with the data in this study.

This study had some merits to be highlighted. It was carried out exclusively with elderly people, a group that has been less evaluated in studies that developed various equations for calculating the GRF estimate. Another merit of the study was the selection of primary care patients, which simulates the condition that represents real life in general outpatient clinics.

In this investigation, the percentage of study patients who had glomerular filtration lower than 60 mL/min was 22.28%, which is equivalent to the national prevalence of CKD in a study in Brazil, which is 21.4% in the elderly^2^. This was not a randomized study.

## CONCLUSION

Creatinine clearance and the C&G equation showed poor performance in predicting GFR estimated by cystatin-C. Both the MDRD study GFR calculation equation and CKD-EPI-Cr can be used as substitutes when cystatin-C dosage is not available, with a slight advantage for CKD-EPI-Cr.

## References

[1] Marinho AWGB, Penha ADP, Silva MT, Galvão TF (2017). Prevalence of chronic kidney disease in adults in Brazil: a systematic review of the literature. Cadernos Saúde Coletiva.

[2] Levey AS, Jong PE, Coresh J, Nahas ME, Astor BC, Matsushita K (2010). The definition, classification and prognosis of chronic kidney disease: a KDIGO controversy report. Kidney Int.

[3] TC Guidelines (2005). Use of cystatin C measurement in the assessment of renal function. Caring for Australians with kidney failure. Nephrology.

[4] Stevens LA, Coresh J, Schmid CH, Feldman HI, Froissart M, Kusek J (2008). Estimating GFR using serum cystatin C alone and in combination with serum creatinine: a pooled analysis of 3,418 individuals with CKD. Am J Kidney Dis.

[5] Stevens LA, Manzi J, Levey AS, Chen J, Deysher AE, Greene T (2007). Impact of creatinine calibration on performance of GFR estimating equations in a pooled individual patient database. Am J Kidney Dis.

[6] DuBois D, DuBois EF (1916). A formula for estimating approximate surface area if height and weight are known. Arch Intern.

[7] Levey AS, Stevens LA, Schmid CH, Zhang YL, Castro AF, Feldman HI (2009). A new equation to estimate glomerular filtration rate. Ann Intern Med.

[8] Inker LA, Schmid CH, Tighiouart H, Eckfeldt JH, Feldman HI, Greene T (2012). Estimating glomerular filtration rate from serum creatinine and cystatin C. N Engl J Med.

[9] Matsushita K, Selvin E, Bash LD, Astor BC, Coresh J (2010). Risk implications of the new CKD epidemiology collaboration (CKD-EPI) equation compared with the MDRD study equation for estimated GFR: the atherosclerosis risk in communities (ARIC) study. Am J Kidney Dis.

[10] Levey AS, Deo A, Jaber BL (2010). Filtration markers in acute kidney injury. Am J Kidney Dis.

[11] Zhang QL, Rothenbacher D (2008). Prevalence of chronic kidney disease in population-based studies: systematic review. BMC Public Health.

[12] Levey AS, Stevens LA, Schmid CH, Zhang YL, Castro AF, Feldman HI (2009). A new equation to estimate glomerular filtration rate. Ann Intern Med.

[13] Stevens LA, Coresh J, Schmid CH, Feldman HI, Froissart M, Kusek J (2008). Estimating GFR using serum cystatin C alone and in combination with serum creatinine: a pooled analysis of 3,418 individuals with CKD. Am J Kidney Dis.

[14] Borges TT, Rombaldi AJ, Correa LQ, Knuth AG, Hallal PC (2012). Prevalence of self-reported morbidity and knowledge about diabetes: a population-based study in a city in southern Brazil. Braz J Kinanthropom Hum Perform.

[15] White SL, Polkinghorne KR, Atkins RC, Chadban SJ (2010). Comparison of the prevalence and mortality risk of CKD in Australia using the CKD epidemiology collaboration (CKD-EPI) and modification of diet in renal disease (MDRD) study GFR estimating equations: the AusDiab (australian diabetes, obesity and lifestyle) study. Am J Kidney Dis.

[16] Jojoa JA, Rivera CE, Delgado EM, Casas HM, Rosas GM, Rosero CY (2017). New ABC classification of chronic kidney disease. Int J Nephrol Renal Fail.

